# Cross-correlation and time series analysis of rabies in different animal species in Nepal from 2005 to 2018

**DOI:** 10.1016/j.heliyon.2024.e25773

**Published:** 2024-02-04

**Authors:** Swochhal Prakash Shrestha, Warangkhana Chaisowwong, Mukul Upadhyaya, Swoyam Prakash Shrestha, Veerasak Punyapornwithaya

**Affiliations:** a Veterinary Public Health and Food Safety Centre for Asia Pacific (VPHCAP), Faculty of Veterinary Medicine, Chiang Mai University, Chiang Mai, 50100, Thailand; b Department of Veterinary Biosciences and Veterinary Public Health, Faculty of Veterinary Medicine, Chiang Mai University, Chiang Mai, 50100, Thailand; c Research Center for Veterinary Biosciences and Veterinary Public Health, Faculty of Veterinary Medicine, Chiang Mai University, Chiang Mai, 50100, Thailand; d Veterinary Epidemiology Section (VES), Department of Livestock Services (DLS), Kathmandu, 44600, Nepal; e National Animal Science Research Institute (NASRI), Nepal Agricultural Research Council (NARC), Lalitpur, 44700, Nepal

**Keywords:** Cross-correlation, Forecasting, Nepal, Rabies, Time series

## Abstract

Rabies is a fatal zoonotic disease, resulting in human and livestock deaths. In Nepal, animal rabies has posed a significant challenge to public health. Because animals are the primary source of rabies in humans, a better understanding of rabies epidemiology in animals is necessary. The objectives of this study were to determine the correlation between rabies occurrences in dogs and livestock animals and to detect the trends and change points of the disease using longitudinal data. The nationwide rabies dataset from 2005 to 2018 was analyzed using cross-correlation, multiple change points, and time series methods. Autoregressive Integrated Moving Average (ARIMA) and Neural Network Autoregression (NNAR) were applied to the time series data. The results show a positive correlation between canine rabies and livestock rabies occurrences. Three significant change points were detected in the time series data, demonstrating that the occurrences were high in the initial years but stabilized before peaking to an upward trend in the final years of the study period. Nonetheless, there was no seasonality pattern in rabies occurrences. The most suitable models were ARIMA (2,1,2) and NNAR (5,1,4) _(12)_. Based on the study findings, both locals and tourists in Nepal need to have enhanced awareness of the potential dangers posed by rabies in canines and livestock. This study offers much-needed insight into the patterns and epidemiology of animal rabies which will be helpful for policymakers in drafting rabies control plans for Nepal.

## 1 Introduction

Rabies is a neglected viral zoonosis, caused by a negative-stranded RNA virus within the Lyssavirus genus of the *Rhabdoviridae* family [1]. The disease affects all warm-blooded mammals, including humans, and develops in the central nervous system, causing encephalitis and ultimately, death [2]. There are two distinct types of rabies in terms of epidemiology: canine-transmitted (urban) rabies and wild animal (sylvatic) rabies [3]. North America and Europe have eliminated urban rabies and are working towards control of sylvatic rabies, while urban rabies is still prevalent in the majority of developing nations in Asia, Africa, and Latin America [[4], [5], [6], [7]]. Roughly 59,000 people in Asia and Africa succumb to rabies every year due to long-standing endemic canine rabies, lack of healthcare facilities for animal bite victims, and inadequate prevention and control strategies [8,9]. The economic burden due to canine rabies is mainly due to premature deaths; however, livestock deaths have also caused considerable economic losses in African countries (Ethiopia, Nigeria, Sudan, and Tanzania) and Asia (China, India, Pakistan, and Bangladesh) [10,11].

Nepal is located in South Asia between India and China, where rabies is highly endemic [[12], [13], [14]]. Furthermore, rabies is listed as one of the prioritized zoonotic diseases in Nepal [15]. Every year in Nepal, the disease claims the lives of between 100 and 200 animals as well as 10 to 100 people [16,17]. A quarter of Nepalis live in areas with a moderate risk of rabies, and nearly half live in high-risk areas while more than 30,000 people and approximately 1000 livestock receive post-exposure treatments annually [18,19]. Among 28,514 animal bite cases reported in Nepal during 2017/18, 26,312 (92 %) were dog bite cases [20]. The agriculture sector engages around 66 % of total population in Nepal, contributing to 1/3rd of GDP with livestock sub-sector contributing 11 % in GDP and approximately about 25.68 % of AGDP. Since Nepal is an agricultural country, animals like goats, cows, and buffaloes are grazed in open fields with freely roaming dogs coming into contact with them, which is believed to be the main mode of transmission for human rabies [21].

There exists a constant risk of rabies spillover from wild mammals to the urban dog (stray and owned) population [22], livestock [23], and humans [24,25]. Most cases of rabies in Nepal are caused by dogs, but even if a dog is immunized, other animals in the area can still spread the disease and maintain the virus cycle in different ecosystems and landscapes [23]. In China, there have been times when the number of people dying from rabies went down temporarily while the number of animals who got it went up [26]. Normally, countries like China [27], Thailand [28], and Tunisia [29] have reported rabies incidence in dogs at a higher rate compared to other livestock and wildlife. In contrast, previous reports from Nepal have shown cattle to be the most affected livestock species followed by dogs [1,30]. Although rabies in dogs can be transmitted to livestock, the exact mechanism by which this occurs is not well understood, resulting in a knowledge gap regarding whether an increase in the number of dog rabies cases correlates with an increase in the number of rabies cases in livestock. Due to the longitudinal structure of the dataset, determining such a relationship requires an analysis that accounts for time series data.

Time series cross-correlation enables the researcher to comprehend the similarity of data between a time series and its lag [31]. Cross-correlation analysis for time series data tracks the movement of two or more sets of time series relative to one another [32]. The utility of cross-correlation analysis in epidemiology lies in its ability to examine the relationship between disease incidence and environmental factors, as well as to survey the spread of disease between populations or geographical locations. Cross-correlation analysis has been used in various epidemiological studies; in Japan to find out if a link exists between home range and the number of SARS-CoV-2 cases [31], in Sri Lanka to study the relationship between the incidence of dengue and climatic factors [33], and in the Netherlands to find out how flu-like illnesses and pneumococcal diseases are related to each other in terms of time [34]. Such analysis could be used to learn more about how rabies spreads between different species in a long study frame. Previously, it has been applied in Bhutan to explore the incidence of rabies cases in dogs and other animals [35].

Several researchers have studied rabies and its epidemiology in Nepal using descriptive data analyses [1,21,30]. For instance, Devleesschauwer et al. conducted a systematic review of the epidemiology, impact, and control of rabies [1]. Furthermore, Dailekh and Kailali were identified as high-risk areas for animal rabies occurrences in Nepal, according to a spatial study conducted by Shrestha et al. (2023) [36]. However, no detailed trend, pattern, or change point analyses have been conducted on the surveillance data to describe the time series of animal rabies occurrences in Nepal. Time series analysis relies on previous data to uncover the underlying time-dependent structure and project future trends [37]. On the other hand, change point analysis detects sudden structural changes in time series analysis, providing factual context for the trend [38]. Recently, numerous epidemiological studies have used a variety of time-series techniques to create prediction models that can be used with a wide range of data [[39], [40], [41]]. Accordingly, autoregressive integrated moving average (ARIMA) and neural network autoregression (NNAR) methods are used together in this study. This approach has been used in animal rabies to discuss trends and predict cases of canine rabies in Thailand [42], cattle rabies in Brazil [43], and the overall animal species in Bhutan [35] and Mexico [44].

Hence, the objectives of our study are: (i) to evaluate the association between reported rabies occurrences in dogs and other livestock animals in Nepal, (ii) to examine rabies occurrences in Nepal over 14 years (2005–2018) for the presence of trends and seasonality using change points and time series methods respectively, and (iii) to compare the prediction performance of time series forecasting models for prospective estimation of rabies occurrences.

## 2 Materials and methods

### 2.1 Data source

The data for this study were obtained from the animal disease epidemiological report maintained by the Veterinary Epidemiology Section (VES). The data included reported rabies cases in animals, both clinical and lab confirmed. The cases are diagnosed by the district veterinary officials based on the epidemiological investigation, history of a dog bite, clinical signs consistent with rabies, and subsequent death of animals, confirmed by the Central Veterinary Laboratory (CVL) with a Fluorescent Antibody Test (FAT). Field samples were not taken in every case, especially when at least one sample from the same herd or location had been confirmed as positive in the lab. This is because rabies is easy to diagnose in a country like Nepal where the disease is common.

### 2.2 Descriptive analysis

The dataset obtained from the VES reported the number of rabies occurrences in different species from 2005 to 2018. A stacked column graph was utilized to give a clear picture of the species-wise distribution.

### 2.3 Cross-correlation analysis

The number of reported incidents involving dogs and livestock species (including cattle, buffalo, goats, sheep, and pigs) was aggregated into monthly series and examined using the cross-correlation function (CCF) in order to determine whether or not a link exists between the two sets of data [35].

The CCF determines whether two time series datasets ($y_{t}$ and $x_{t}$) on terms $y_{t}$ correlate with the past lags of the x series. The values of lag can range from zero, positive, or negative (e.g., 0, ±1, ±2, ±3, …, ±n). The positive value of h indicates a correlation between the x variable at a time after t defined as $x_{t+h}$ and the y variable at time t*.* Alternatively, the negative value for h indicates a correlation between the x variable at a time before t denoted as $x_{t-h}$ and the *y* variable at time t. For instance, if a lag number is zero, it reveals that $y_{1},y_{2},y_{3},...,y_{n}$ correlate with $x_{1},x_{2},x_{3},...,x_{n}$. While a lag number equal to 1 suggests that $x_{1},x_{2},x_{3},...,x_{n}$ correlate with $y_{2},y_{3},y_{4}...,y_{n}$. If a lag number is −1, it shows that $x_{2},x_{3},x_{4}...,x_{n}$ correlate with $y_{1},y_{2},y_{3},...,y_{n}$.

In this study, the occurrences of rabies in dogs (x variable) were examined to establish whether they lead to rabies in other domestic animals (y variable). Since the average incubation period of rabies is six months, the CCFs were estimated for lag windows of up to six months [45]. The CCF function in R statistical software was used to perform the analysis. The level of statistical significance for CCF analysis was set as α = 0.05.

### 2.4 Change point analysis

To identify significant changes in rabies occurrences during the period from 2005 to 2018, change point analysis was applied to the obtained dataset [38,46,47]. These occurrences were assumed to follow the Poisson distribution due to the count nature of the data.

Given the m segments of the time series rabies occurrences data, a binary segmentation technique was utilized for change point detection with following function [47]:

$$\begin{array}{c} \sum_{i=1}^{m+1}\left[C\left(x_{\left(t_{i-1}\right):t_{i}}\right)\right]+\beta f\left(m\right) \end{array}$$

where C is a function cost of a segment and βf(m) is a penalty to guard against overfitting.

The *cpt.meanvar* function from the “change point” package, offering a binary segmentation approach, was used to detect changes in both mean and variance of the time series rabies data. Initially, the *cpt.meanvar* algorithm identified a single change point within the dataset. After identifying the first change point, the data were split into two subsegments at the location of the change point. In both datasets, the single change point process was repeated. In the case of additional change points being detected, the data was then divided into additional subsegments. This process was repeated until there were no change points in the subsegments [47].

### 2.5 Seasonality test

The Ollech-Webel overall seasonality test (WO-test) using the “seatests” package was used to check for seasonality in the time series dataset. The WO-test combines the results of the QS test and the KW (Kruskal-Wallis) test. The QS test determines seasonality by examining the autocorrelation of seasonal lags while the KW test uses a non-parametric rank-based approach to test the monthly data [48]. If the p-value of the QS-test is below 0.01 or the p-value of the KW-test is below 0.002, the WO-test indicates seasonality in the data. The seasons were classified as per the guidelines of the reporting format of the Veterinary Epidemiology Section: summer (June, July, August), autumn (September, October, November), winter (December, January, February), and spring (March, April, May).

### 2.6 Time series analysis

For time series analysis, the data were analyzed using the following steps: (i) decomposing data into trend and seasonality, (ii) identifying the most suitable form of time series models (ARIMA and NNAR), and (iii) comparing predictive performance of the most suitable models from the ARIMA and NNAR based on training and validation datasets.

Data on animal rabies occurrences from 2005 to 2018 (n = 1085) were used to create the time series model. The data was divided into two parts; data from 2005 to 2017 (training data) and 2018 (validation data) for model development and validation and evaluation of the model's predictive effects, respectively.

### 2.7 Decomposition of time-series data

Additive decomposition was used to evaluate the seasonality and trend of animal rabies occurrences. This method decomposes a time series dataset into three parts: trend, seasonality, and remainder component on a 12-month basis. Equation (2) depicts an additive decomposition;

$$Y_{t}=T_{t}+S_{t}+R_{t}$$

where $Y_{t}$ is the number of animal rabies occurrences, $T_{t}$ is the trend-cycle component, $S_{t}$ is the seasonal component, and $R_{t}$ is the remainder component, all at period t.

### 2.8 Time series forecast model development

The data from January 2005 to December 2017 (156 months) was used to create the time series model using ARIMA and NNAR methods. ARIMA is a generalized model of Autoregressive Moving Average (ARMA) that the combines the Autoregressive (AR) and Moving Average (MA) processes and builds a composite model of the time series [49]. The expression of ARIMA is given as

ARIMA(p,d,q)

where *p* = order of autoregression, *d* = degree of trend difference, *q* = order of MA.

NNAR are mathematical models using lagged values of time series as input into a neural network. A network of three function layers is joined together by acyclic linkages [50]. For seasonal data, the NNAR model can be written as

$${NNAR\left(p,P,K\right)}_{m}$$

where *p* = the last observed values from the same season used as inputs, *P* = lagged inputs, *K* = number of neurons (nodes) in the hidden layer, and *m* = the number of months.

### 2.9 Forecast and model performances

The performance of time series models was tested to evaluate how well those obtained from the training dataset could predict the unseen follow-up data (testing dataset). Thus, time series models developed from training datasets were used to predict occurrences in 2018. Model prediction accuracy was assessed by comparing the forecast and actual occurrences of animal rabies. Root mean squared error (RMSE), mean absolute error (MAE), and mean absolute scaled error (MASE) were used as evaluation error metrics to determine prediction performances among the developed models [50]. The values of all error metrics were calculated for the in-sample (training data), and also used to evaluate how well the models created from the training data performed on the hold-out sample (validation data). The model was generally agreed to be better if the error metrics were lower [42,51].

R statistical software and “xts”, “tsbox”, “ggplot”, “TSstudio”, “forecast”, and “forecast Hybrid” packages were used to perform data analysis and time series modeling [52].

## 3 Results

### 3.1 Descriptive analysis

**Fig. 1:**
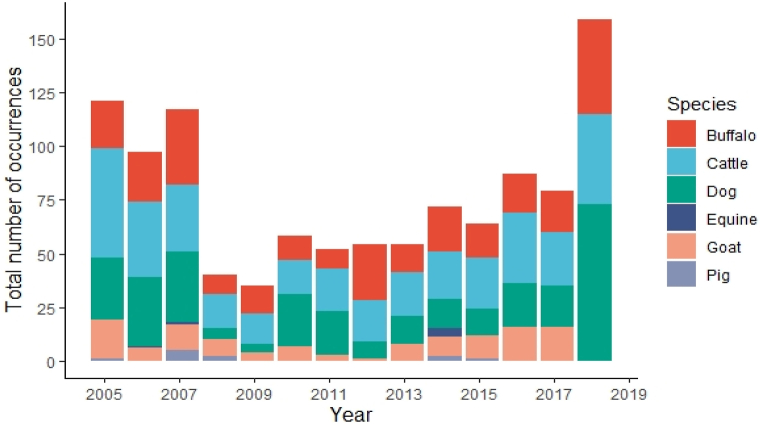
Species-wise distribution of animal rabies occurrences in Nepal from 2005 to 2018 as reported in the Veterinary Epidemiology Section.

Fig. 1 portrays the species-wise distribution of animal rabies occurrences. On the whole, rabies was most often seen in cattle, followed by dogs. In Nepal, animal rabies cases were also reported in other livestock species such as buffalo, goats, and pigs.

### 3.2 Cross-correlation analysis

**Fig. 2:**
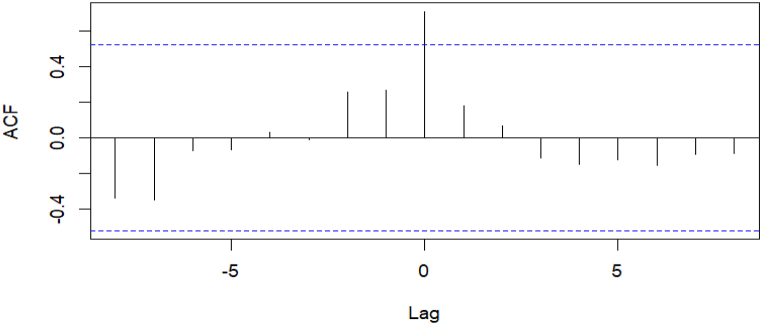
Autocorrelation plot between cases of rabies in dogs and domestic animals reported between January 1, 2005, and December 31, 2018, in Nepal, lagged by six months.

Fig. 2 shows a statistically significant positive cross-correlation between the number of rabies cases in dogs and other domestic animals at a monthly lag of zero. This implies that as the number of rabies occurrences in dogs increased, so did that in other domestic animals during the same month. In other words, there was no lag in domestic animal occurrences of rabies following the spike in canine rabies.

### 3.3 Change point analysis

The time series data on the number of rabies occurrences indicated three change points. Following the identification of the change points, their corresponding segments represent the mean number of rabies occurrences observed during the period. After the first change point, the number of occurrences remained stable for five years (2008–2013). The second change point revealed a steady increase in rabies occurrence while the third change point depicted a sharp upward trajectory.

### 3.4 Seasonality

According to the results of the WO-test (p = 0.637), no seasonality pattern was revealed in the animal rabies time series dataset.

### 3.5 Trends

The classical additive decomposition plot for animal rabies occurrences (Fig. 4) showed a peak in the first few years (2005–2008) after which there was a downward trend. From late 2010 to early 2016, animal rabies occurrences appeared to be stable. Subsequently, there was an upward trend in the number of animal rabies occurrences. The seasonal pattern demonstrated that the peak of animal rabies occurrences was mainly observed in the first few months of the calendar year (January–February) and showed a gradual decline, followed by a smaller second peak in the incidence rate during the middle of the year. The random component showed varying residuals with observed large values over the final years.

### 3.6 Fitted time series model performances

Fig. 5 depicts a graphical representation of the actual and fitted rabies occurrences based on the ARIMA and NNAR models from the training set with the testing set forecast based on the validation data. During fitting of the time series model, ARIMA (2,1,2) and NNAR (5,1,4) [12] best represented the dataset under study. Moreover, the predicted values for both models were low in comparison to the observed real values.

The error matrices from time series models based on RMSE, MAE, and MASE are presented in Table 1. The NNAR was found to perform better than ARIMA when used with training data. Also, NNAR outperformed ARIMA in the validation dataset.

**Table 1:** Error matrices for time series models applied to the training and validation datasets.

Model	Training Data (Data: 2005–2017)			Validation Data (Data: 2018)		
	RMSE	MAE	MASE	RMSE	MAE	MASE
ARIMA	4.30	3.44	0.72	9.21	8.42	1.75
NNAR	2.55	1.99	0.42	9.15	8.40	1.74

## 4 Discussion

The results of this study revealed an erratic trend in the animal rabies dataset with three distinct change points and no seasonality. A positive correlation between dog and livestock rabies occurrences was also discovered. Additionally, the forecast performance of two different models was comparable with the actual values.

Overall, dogs and cattle accounted for the majority of reported cases but the cases in cattle outnumbered those in dogs and other animals (Fig. 1). Cattle have greater economic value in an agricultural country like Nepal [53] which may be the reason for the cases of cattle being reported more frequently than other animals. According to the reports, no documentation on wildlife rabies exists in Nepal and hence, dogs were the source of spillover infections in cattle, other domestic animals, and occasional infections in humans. There are no exact reports regarding the stray dog population in Nepal, but it is estimated to be nearly two million, equivalent to one dog per 10 humans [1]. This study also attempts to elaborate on the relationship between dog cases and other domestic animals since dogs are the reservoir and vector of rabies transmission. According to the evidence obtained for this study, there has been an increase in livestock rabies occurrences at the same time as canine rabies. An earlier study in Namibia revealed similar findings, with black-backed jackals predicting rabies in dogs and domestic animals [45], while in Bhutan, a significant correlation was demonstrated between rabies in dogs and other domestic animals [35]. In canine rabies-endemic countries like Nepal, this relationship may have a significant impact on livestock. The annual cost of livestock losses due to rabies is estimated to be 12.3 million US dollars in canine endemic regions of Asia and Africa [54]. This association further substantiates the need for enhancing rabies surveillance in the event of rabies occurrence in dogs. In terms of public health awareness, since stray dogs roam around freely, one dog can infect multiple other animals which might be the reason for a simultaneous increase in the number of rabies cases in livestock when there are cases in dogs. Thus, people must be made aware of the risk carried by livestock rabies, especially tourists and foreigners coming to Nepal.

Generally, the trend is non-linear with no specific pattern during the study period which aligns with previous reports [55]. Early in the study period, there is a high number of occurrences before the first change point which can be attributed to the absence of rabies control programmes such as mass anti-rabies vaccination and animal birth control in dogs [21]. In 2008, the Rabies in Asia Foundation organized a conference to discuss various strategies for eradicating rabies in Nepal. Various animal welfare organizations like Kathmandu Animal Treatment (KAT) Centre, Animal Nepal, and the Global Alliance for Rabies Control (GARC) with help from the Nepal government have organized free anti-rabies vaccination campaigns and actively participated in the rabies control programs in Nepal, initiating the celebration of World Rabies Day [56]. The downward trend, followed by a stable number of occurrences for a few years after 2008 could be attributed to such programs in the second segment before the second change point. Nevertheless, there was a rise in the number after the third change point late in the study period which could be attributed to the adoption of the “Zero by 30” goal after 2016 to eliminate human rabies, while the passive rabies surveillance system for animal rabies in Nepal improved the frequency of reporting the rabies cases [57].

**Fig. 3:**
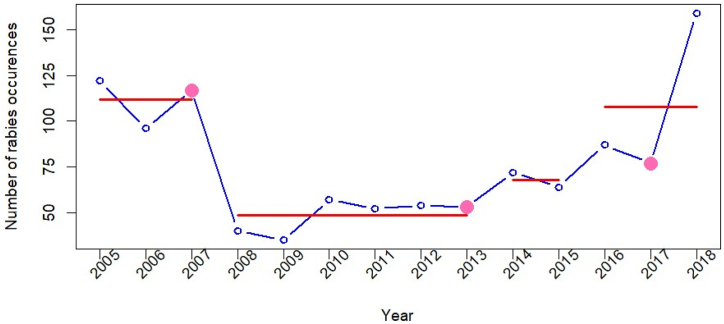
Change points in time series for rabies occurrences reports from 2005 to 2018. Pink dots are change points and red lines are the corresponding segments. (For interpretation of the references to colour in this figure legend, the reader is referred to the Web version of this article.)

Rabies occurrences were reported throughout the year with more being reported in spring (January–February) as shown in Fig. 3. This finding is in agreement with a previous report on Nepal in which more rabies cases were reported during February compared to other months [30]. The increased incidence of rabies during spring can be connected to the breeding season of dogs since various studies have conjectured that increased contact rates between dogs during mating behavior led to the risk of virus transmission [[58], [59], [60]]. There has been evidence of seasonality seen in rabies cases in Mexico [44], Thailand [60], and China [61]. Although some months of the year exhibited a higher number of occurrences compared to others, the WO-test indicated a total absence of seasonality. This result was comparable to a study undertaken in Brazil which reported an absence of seasonality for cattle rabies from 2006 to 2013 [43].

**Fig. 4:**
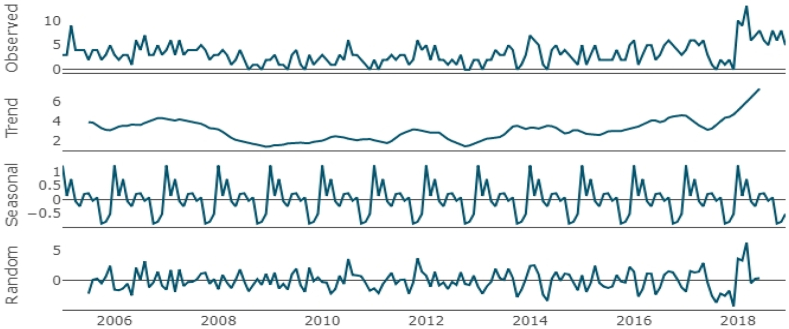
Decomposition of the number of time series animal rabies occurrences from 2005 to 2018 into actual (observed), trend, decomposed seasonal trait (seasonal), and random fluctuation.

**Fig. 5:**
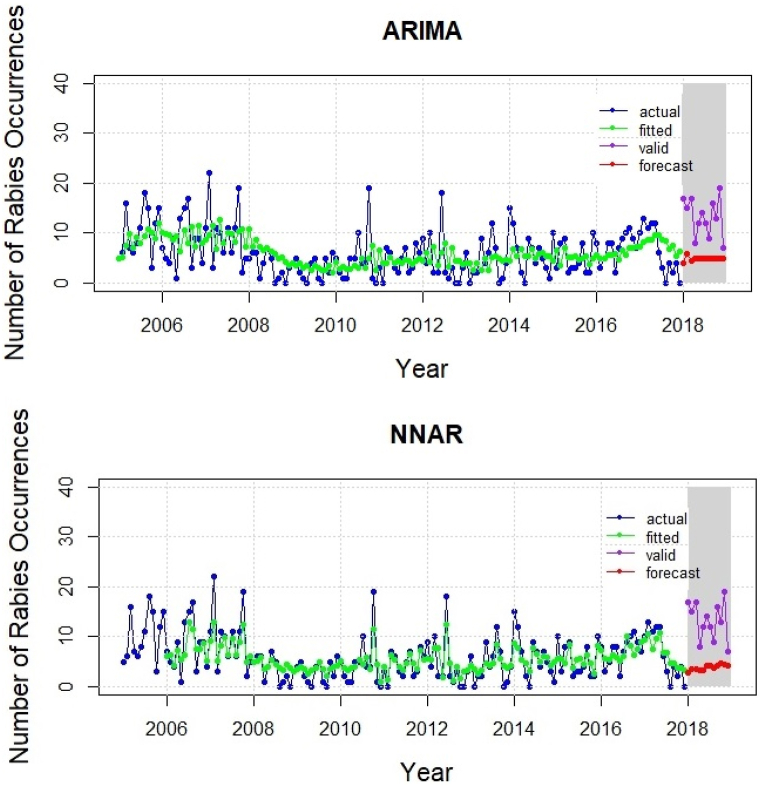
Actual, fitted, validated, and forecast values from ARIMA and NNAR models.

In the event of no seasonality pattern, this study has scouted the use of ARIMA and NNAR for time series modeling [62]. Furthermore, the findings of this study reveal that NNAR outperformed ARIMA, possibly due to the advantage NNAR carries in exhibiting non-linear characteristics of rabies occurrences [51,63]. Nevertheless, the final model is likely to be revised whenever data are brought up to date. It is generally agreed that in order to produce more accurate results, the forecast should be created using the most recent data. Since the data for this study from 2005 to 2018 were the most recent data available, the forecast should be projected for 2019 and subsequent years. The updated data from 2005 to 2023 should be used if the forecasts for 2024 and subsequent years are to be targeted. At this point, policymakers should be urged to use time series analysis to predict future occurrences of rabies using the most recent data. Additionally, the analysis could be performed annually to make changes based on the forecast.

Despite these findings, there are a few limitations to this study. Firstly, the dataset utilized was an accumulation of monthly reports from all the districts in Nepal which may be subjected to underreporting or minor differences over the years. Secondly, the study does not identify temporary patterns in rabies cases at the district level since the data would contain a significant amount of zero values which would be inappropriate for the models used. Also, the study does not include a human rabies dataset since this is not publicly available with permission required for access, limiting the comparison between rabies cases in humans and animals. However, the current study focuses on gaining insight into animal rabies, and future studies can attempt to establish a relationship between human and animal rabies cases.

In spite of the earliest rabies elimination committee being established since 1979 in Nepal, no realistic and systematic national rabies control policy and strategy has been developed by the Government of Nepal (GoN) [56]. The complexity of zoonotic diseases like rabies makes management and prevention more difficult; hence, a multi-sectoral data-driven One Health Approach may be a better strategy for combating rabies. In a resource-limited and developing country like Nepal, any control strategy formulated must be efficient and sustainable. Thus, our findings will help stakeholders to develop control strategies for animal rabies and if similar research is carried out simultaneously on the human rabies dataset, there will be an absolute plan that will help in the embarkment of the ‘Zero by 30’ goal adopted by the world. For further research with similar datasets, we suggest inclusion of variables such as average temperature, dog population density in each area, and vaccination history that would improve the predictability of the developed model.

## 5 Conclusions

In summary, the analysis presented in this study indicates a stable trend from 2010 to 2015 with an increase in cases from 2016 to 2018. There is also an absence of seasonality in the occurrence of rabies. In addition, a significant correlation between canine rabies and rabies among livestock is observed. The NNAR is a better model than the other forecast models used in the study. To the best of researchers’ knowledge, this is the first study to use time series models to describe trends and seasonality while also investigating the correlation of occurrences between species. Urban animal rabies control is the key to attaining the zero by 30 goal to eliminate human deaths by rabies. For the development of control strategies, similar time series approaches could be applied to the latest dataset by utilizing different collaborating stakeholders to predict the future trends of animal rabies and allocate the necessary resources accordingly.

## Ethics approval

This study utilizes a secondary data of rabies surveillance system maintained under Department of Livestock Services (DLS), Nepal; therefore, ethical approval for animal or human uses is not required. For this study, we have permission to use data from the DLS (Reference number: 83/PCU/2022).

## Funding statement

This study was funded by 10.13039/501100002842Chiang Mai University, Chiang Mai, Thailand (Grant: R66IN00356 and Grant: R000029530/FF66/021).

## Data availability statement

The data used in this study is accessed from Veterinary Epidemiology Section approved by the Department of Livestock Services (DLS), Ministry of Agriculture and Livestock Development, Nepal. An official permission letter is required for the use of data. The data used in this study was approved by the DLS (Reference number: 83/PCU/2022).

## CRediT authorship contribution statement

**Swochhal Prakash Shrestha:** Writing – review & editing, Writing – original draft, Software, Methodology, Formal analysis, Conceptualization. **Warangkhana Chaisowwong:** Writing – review & editing, Writing – original draft, Project administration, Conceptualization. **Mukul Upadhyaya:** Writing – review & editing, Validation, Resources, Investigation, Data curation. **Swoyam Prakash Shrestha:** Writing – review & editing, Validation, Resources, Investigation, Data curation. **Veerasak Punyapornwithaya:** Writing – review & editing, Writing – original draft, Visualization, Validation, Supervision, Software, Resources, Project administration, Methodology, Funding acquisition, Formal analysis, Data curation, Conceptualization.

## Declaration of competing interest

The authors declare that they have no known competing financial interests or personal relationships that could have appeared to influence the work reported in this paper.
